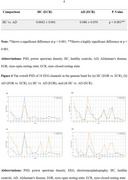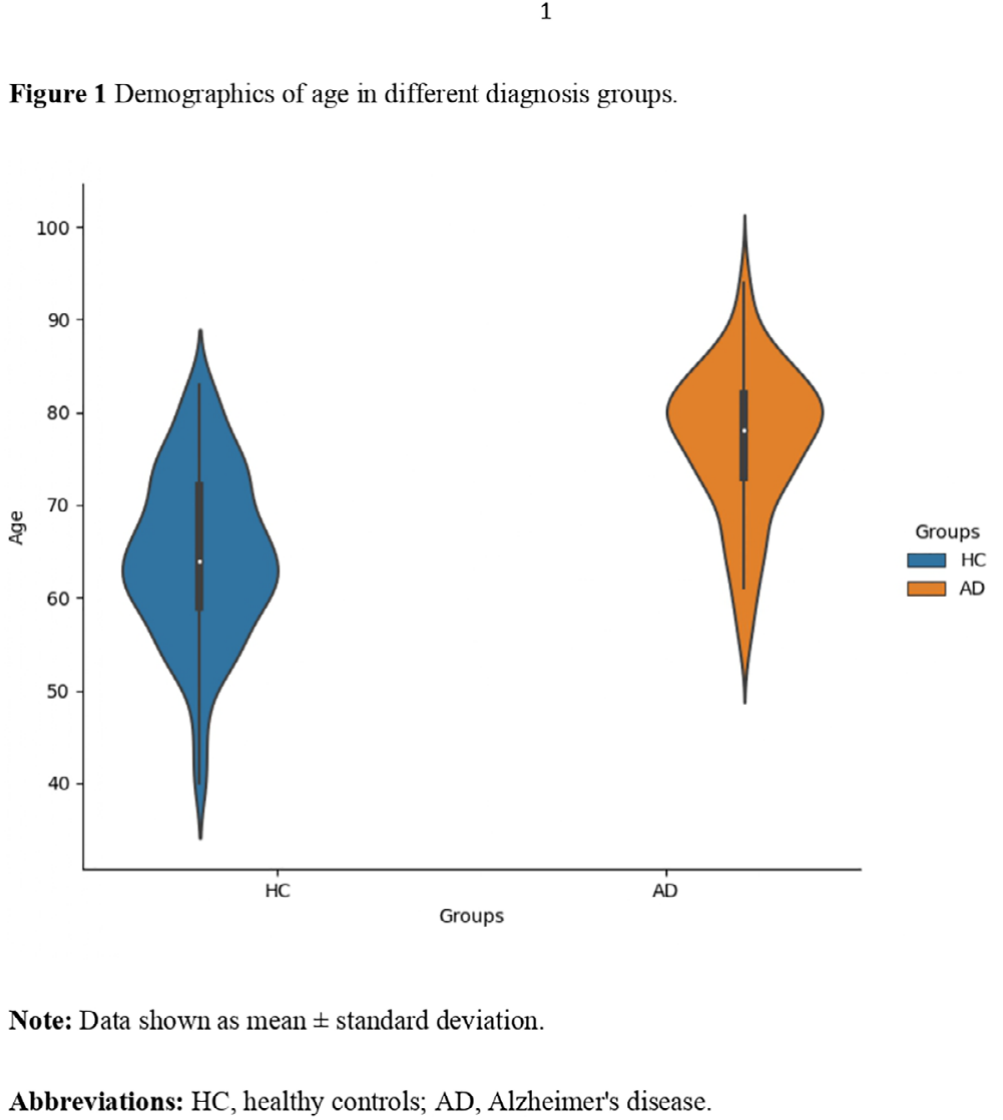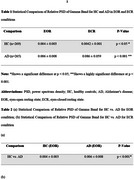# Investigating Gamma Frequency Band PSD in Alzheimer's Disease Using qEEG from Eyes‐Open and Eyes‐Closed Resting Conditions

**DOI:** 10.1002/alz70856_098118

**Published:** 2025-12-24

**Authors:** Chanda Simfukwe, Seong Soo A An, Young Chul Youn

**Affiliations:** ^1^ Gachon University, Seongnam‐Daero, Korea, Republic of (South); ^2^ Gachon university, Seongnam, Korea, Republic of (South); ^3^ Chung‐Ang University Hospital, Seoul, Korea, Republic of (South)

## Abstract

**Background:**

Alzheimer's disease (AD) is a significant brain disorder commonly linked to aging, characterized by various neuronal changes that affect resting state electroencephalography (rEEG) patterns. Many studies have reported alterations in delta, theta, alpha, and beta power, while relatively few have examined gamma activity's rEEG. This study aimed to analyze the changes in power spectrum density (PSD) of gamma oscillations in eyes‐open resting (EOR) and eyes‐closed resting (ECR) EEG of AD patients compared to healthy controls (HC).

**Method:**

This study utilized rEEG data from 534 participants aged 40‐90, including 269 HC and 265 individuals with AD. The quantitative EEG (qEEG) analyses were conducted separately for HC and AD groups in EOR and ECR states to analyze the PSD gamma band range 30‐100 Hz (Welch method), coherence analysis, and functional connectivity. The rEEG data were preprocessed using EEGlab and Brainstorm toolboxes within MATLAB R2020b, and statistical analyses were performed using ANOVA.

**Result:**

The PSD analysis revealed a significant increase in the gamma frequency band across 19 EEG channels, particularly in the AD group's frontal and temporal brain regions, compared to the HC group during EOR and ECR states. At the source level, the gamma power was elevated in the AD group but decreased in the HC group, with a highly significant difference in the ECR. Additionally, the pairwise coherence analysis and functional connectivity between different brain regions was significantly elevated in the AD group during the ECR state but decreased in the EOR state compared to HC subjects.

**Conclusion:**

These findings provide valuable insight into the progression of AD and underscore the importance of the recent 30‐90 Hz gamma band research. This frequency band shows potential as a cost‐effective and comprehensive biomarker for differentiating individuals with AD from HC in dementia research.